# Assessing the role of mitonuclear interactions on mitochondrial function and organismal fitness in natural *Drosophila* populations

**DOI:** 10.1093/evlett/qrae043

**Published:** 2024-08-09

**Authors:** Stefano Bettinazzi, Jane Liang, Enrique Rodriguez, Marion Bonneau, Ruben Holt, Ben Whitehead, Damian K Dowling, Nick Lane, M Florencia Camus

**Affiliations:** Department of Genetics, Evolution and Environment, University College London, London, United Kingdom; Department of Genetics, Evolution and Environment, University College London, London, United Kingdom; Department of Genetics, Evolution and Environment, University College London, London, United Kingdom; Department of Genetics, Evolution and Environment, University College London, London, United Kingdom; Department of Genetics, Evolution and Environment, University College London, London, United Kingdom; Department of Genetics, Evolution and Environment, University College London, London, United Kingdom; School of Biological Sciences, Monash University, Melbourne, VIC, Australia; Department of Genetics, Evolution and Environment, University College London, London, United Kingdom; Department of Genetics, Evolution and Environment, University College London, London, United Kingdom

**Keywords:** mitochondria, mitonuclear interactions, *Drosophila melanogaster*, OXPHOS, fitness, local adaptation

## Abstract

Mitochondrial function depends on the effective interactions between proteins and RNA encoded by the mitochondrial and nuclear genomes. Evidence suggests that both genomes respond to thermal selection and promote adaptation. However, the contribution of their epistatic interactions to life history phenotypes in the wild remains elusive. We investigated the evolutionary implications of mitonuclear interactions in a real-world scenario that sees populations adapted to different environments, altering their geographical distribution while experiencing flow and admixture. We created a *Drosophila melanogaster* panel with replicate native populations from the ends of the Australian east-coast cline, into which we substituted the mtDNA haplotypes that were either predominant or rare at each cline-end, thus creating putatively mitonuclear matched and mismatched populations. Our results suggest that mismatching may impact phenotype, with populations harboring the rarer mtDNA haplotype suffering a trade-off between aerobic capacity and key fitness aspects such as reproduction, growth, and survival. We discuss the significance of mitonuclear interactions as modulators of life history phenotypes in the context of future adaptation and population persistence.

## Introduction

Metabolism lies at the core of life history theory (Burger et al., 2019). To thrive, organisms must adapt and exploit the resources offered by the environment. Mitochondria are key for metabolic adaptation, as they are central hubs for both energy transduction and intermediary metabolism in eukaryotes—a major determinant of all aspects of fitness, including growth, development, and reproductive success (Lane, 2009). Despite its key role, oxidative phosphorylation (OXPHOS) is uniquely vulnerable to disruption, as it depends on components encoded by two different genomes, mitochondrial (mt) and nuclear (n), which must interact harmoniously with each other to preserve bioenergetic efficiency (Blier et al., 2001; Dowling et al., 2008; Lane, 2009, 2011; Wolff et al., 2014). This implies that coevolution between both genomes is key to sustaining bioenergetic function since mitonuclear interactions are perpetually stressed by their very different evolutionary dynamics. As the mitochondrial genome is typically uniparentally inherited and mutated at a faster rate than the nuclear genome across many species, it is predicted that the recombining nuclear genome will respond adaptively to its fast-changing mitochondrial counterpart (Barreto et al., 2018; Healy & Burton, 2020; Hill, 2020). This process has been shown to result in tight mitonuclear coevolution in some taxa and has the potential to drive rapid divergence between populations in their mitonuclear genotypes (Biot-Pelletier et al., 2023; Burton, 2022; Morales et al., 2018; Wang et al., 2021). Given emerging evidence for the need of intergenomic matching (Lane, 2011; Latorre-Pellicer et al., 2016; Ma et al., 2016; Rank et al., 2020), admixture events between disjunct populations with unique trajectories of mitonuclear coevolution might therefore present a challenge, as they could generate genomic incompatibilities.

Environmental changes are occurring at an unprecedented pace, with large consequences for species persistence and distribution (Outhwaite et al., 2022). Many species are predicted to have declining or altered distributions, requiring adaptation to new environments or migration to more favorable habitats. This process could lead to divergent populations having to reunite and share a common niche. Although new mitonuclear variants may foster evolutionary innovations and be favored under certain conditions (e.g., providing better adaptation to the environment), admixture may also unmask mitonuclear incompatibilities. These incompatibilities can manifest as fitness deficits in life history traits, with examples found in yeast (Biot-Pelletier et al., 2023; Lee et al., 2008), as well as across multicellular eukaryotes including plants (Levin, 2003), invertebrates (Burton et al., 2006; Ellison & Burton, 2006; Ellison et al., 2008; Meiklejohn et al., 2013; Niehuis et al., 2008; Rank et al., 2020; Sackton et al., 2003; Zhang et al., 2017), and vertebrates (Barrientos et al., 1998; Dey et al., 2000; Ma et al., 2016; Moran et al., 2024).

The colonization of *Drosophila melanogaster* into the Australian continent is recent and traces back just a few hundred years (Adrion et al., 2015; Hoffmann & Weeks, 2007). Despite continued admixture between populations (Bergland et al., 2016), the Australian eastern latitudinal cline has remained stable over the decades; it is defined by predictable genetic and phenotypic variation suggesting enduring climatic selection (Adrion et al., 2015; Camus et al., 2017b; Chakraborty et al., 2020; Hoffmann & Weeks, 2007; Lajbner et al., 2018; Sgro et al., 2010). Prior research has revealed the existence of two main mitochondrial haplogroups segregating along the Australian east coast, distinguished by 15 SNPs, whose frequency clines in opposing patterns (Camus et al., 2017b). While Haplogroup A is most commonly found in the north (60%–100% frequency in top three northern populations samples), this frequency decreases to 20%–30% in southern populations where Haplogroup B is more common. Little genetic variation has been detected within each haplogroup, with one dominant haplotype (haplotypes A1 and B1), and these haplotypes have been suggested to contribute to local adaptation in fly populations. For instance, the haplotype predominant in the north of the cline has been found to confer heat tolerance to flies in an isogenic nuclear background, whereas the haplotype predominant in the south confers cold tolerance (Camus et al., 2017b).

Here, we took advantage of this model system to examine the contribution of mitonuclear genotypes to locally adaptive phenotypes. Using subtropical and temperate *Drosophila* populations from the ends of the Australian east-coast cline, we created a full-factorial panel of populations comprising two putatively matched (Townsville “tT”; and Melbourne “mM”) and two putatively mismatched mitonuclear combinations (“mT” and “tM”). Given the A1 haplotype is predominant in Townsville and B1 is predominant in Melbourne, we assigned Townsville populations carrying A1 as the “predominant mitonuclear combination,” and those with the B1 haplotype as the “rarer mitonuclear combination.” Similarly, Melbourne populations with B1 are termed predominant, and those with A1 are rarer. For clarity of nomenclature, in this article, we have renamed the A1 and B1 haplotypes as “t” and “m.” Using these lines, we then test the prediction that “t” and “m” haplotypes exhibit signatures of coevolution with Townsville “T” and Melbourne “M” nuclear backgrounds, respectively, whereby the predominant (putatively matched) mitonuclear combinations exhibit superior bioenergetic and life history performance relative to the rarer (putatively mismatched) combinations.

We explored the physiological impact of mitonuclear mismatch by assessing effects on mitochondrial physiology in tandem with metabolically important life history traits. Mitochondrial phenotyping was achieved through high-resolution respirometry, which pinpoints the respiratory complex(es) or pathway(s) that may be impacted by mitonuclear disruption. We examined: (i) substrate-specific oxygen consumption and reactive oxygen species (ROS) flux, linked with the activity of the different OXPHOS complexes and pathways; (ii) maximal coupled and uncoupled respiration; and (iii) standalone activity of the final oxidase of the electron transport system (cytochrome *c* oxidase). These metabolic measurements were coupled with a suite of life history trait phenotyping, including sex-specific fitness, development time, locomotory activity, and thermal tolerance. Our results suggest that mitonuclear mismatching may impact the organismal phenotype since mismatched cybrid populations exhibited an apparent increase in key respiratory components but a decrease in fitness components relative to one or both matched populations. Overall, our study supports the idea that intergenomic interactions can be a strong determinant of individual fitness, adaptive capacity, and population persistence.

## Materials and methods

### Mitonuclear panel

Two replicated *Drosophila melanogaster* populations were sourced from the Australian east-coast cline in early 2021. These populations were from Townsville “T” (latitude: −19.26, longitude 146.81) and Melbourne “M” (latitude: −37.77, longitude: 144.99). Following the creation of these massbred populations, a full-factorial mitonuclear panel was generated. The panel followed a two-letter nomenclature, indicating the mitochondrial genome first and the nuclear background second (lower and uppercase letters, respectively). It included two populations with predominant and putatively matched mitonuclear genotypes, named after the nDNA sampling area (i.e., “tT”—Townsville and “mM”—Melbourne), and two rarer, putatively mismatched cybrid populations (“mT” and “tM”), where the mitochondrial and the nuclear background were reciprocally swapped, using a balancer chromosome crossing scheme (Supplementary Figure S1) (Clancy, 2008). For simplicity, the mitonuclear populations will now be referred to as “matched” and “mismatched” populations throughout the manuscript. Haplotypes “t” and “m” have been referred to as haplotypes “A1”’ and “B1” in previous studies (Camus et al., 2017b; Lajbner et al., 2018). Detailed protocols are provided as *electronic*Supporting Material.

Experimental lines were maintained at standard laboratory conditions (25 °C, 50% RH, 1:1 protein:carbohydrate P:C diet, 12:12 light:dark day cycle). Mitochondrial DNA congruence was routinely checked for by means of PCR, whereas nuclear genetic variance was preserved by regular backcrossing to the nuclear-correspondent massbred native lines. Prior to each experiment, flies were reared in density-controlled conditions (20 eggs per vial), sorted by sex 48 hours posteclosion (excluding reproductive performance), let to acclimate in new food vials and finally assayed at 4–7 days of age. Reproductive performance assays used the same rearing scheme for focal flies; however, experimental flies were collected as virgin (within 2–5 hr posteclosion).

### Mitochondrial physiology

Mitochondrial bioenergetics were characterized at 25 °C on fly permeabilized tissue using dedicated Oxygraph-2k-FluoRespirometers (Oroboros Instruments, Innsbruck, Austria), following existing protocols with minor modifications (Bettinazzi et al., 2019; Gnaiger, 2020; Rodríguez et al., 2021, 2023). Following a specific SUIT protocol (see Supporting Information), we assessed mitochondrial respiration sustained by different combinations of respiratory complexes and in different respiratory states. This included the activity of complex I (CI), proline dehydrogenase (ProDH), complex II (CII), glycerophosphate dehydrogenase (GpDH) and complex IV (CIV), as well as respiratory states such as Leak (nonphosphorylating resting state, state 4 or 2ʹ), OXPHOS (coupled respiration, state 3) and ETS (uncoupled respiration, state 3u). H_2_O_2_ fluxes were evaluated in parallel with respiratory rates, and parameters were named accordingly. Respirometry data were expressed as O_2_ fluxes normalized for tissue mass (pmol O_2_ ∙ s^−1^ ∙ mg^−1^) and as flux control ratios (FCR), normalized for maximum coupled respiration (CI + ProDH + CII + GpDH_P_) (Gnaiger, 2020). Change in respiration following the addition of specific substrates or inhibitors was expressed by means of flux control factors. Oxygen flux parameters were also condensed by means of a principal component analysis. ROS rates were expressed as H_2_O_2_ fluxes normalized for tissue mass (pmol H_2_O_2_ ∙ s^−1^ ∙ mg^−1^) or as ratios, in function of the step-specific oxygen consumption (H_2_O_2_ ∙ O_2_^−1^).

### mtDNA copy number

Genome abundance was determined fluorometrically on a Mastercycler RealPlex thermocycler (Eppendorf, DE), using the KAPA SYBR FAST qPCR Master Mix Kit (KAPABIOSYSTEMS) and two complementary sets of primers, respectively amplifying *cox1* (mitochondrial) and *rosy* (nuclear) genes. For both genes, the cycle thresholds (CT) were measured in duplicates, and the mtDNA copy number relative to the nuclear genome was determined by the formula (2^−ΔCT^)0.2, with ΔCT referring to the difference between the mitochondrial and the nuclear gene mean CT (Ballard et al., 2007b).

### Locomotor activity

Fly locomotor activity was recorded for 48 hr using dedicated Drosophila Activity Monitors (DAM2, Trikinetics) and calculated as the number of counts (infrared beam breaks) per minute (Anderson et al., 2022). The activity was then condensed into 30 minutes activity and further in timeframe-specific activity (i.e., dawn, day, dusk, and night).

### Reproductive performance

Reproductive fitness was investigated in both female and male individuals, the latter in both a noncompetitive and competitive environment. All adults were collected as virgins (within 5 hr posteclosion), reared and acclimated at the same standard laboratory conditions (25 °C, 50% RH, 1:1-P:C diet, 12:12 light:dark cycle) and of the same age. For female fitness, experimental females were given the opportunity to mate with standard LHm (Larry Harshman, moderate density population) (Rice et al., 2005) males for 5 hr at a concentration of 30 flies per vial (1:1 sex ratio). After mating, females were placed in separate vials to lay eggs for a period of 14 hr. Female fecundity (number of eggs laid; *n* eggs · female^−1^), fertility (adult offspring produced from those eggs; *n* adults · female^−1^) and eggs to adults survival ((adults · eggs^−1^) %) were then measured (Camus et al., 2017a, 2020b). For male noncompetitive fitness, experimental males were given the opportunity to mate with standard females from the massbred LHm population for 5 hr at a concentration of 30 flies per vial (1:1 sex ratio). Females were then sorted into individual vials and allowed to lay eggs for 48 hr. They were then transferred to new vials and left to oviposit for an additional 48 hr. Male fertility (adult offspring from the 96-hr lay; *n* adults · female^−1^) was then measured (Camus et al., 2020a). For male competitive fitness, a trio of experimental males competed with a trio of LHm *bw-* males (outbred population with homozygous recessive brown eye mutation) for the mating of six LHm *bw-* virgin females over a period of 24 hr. Red-eyed (wild type; WT) progeny was assigned to the experimental line, while brown-eyed progeny to the competitor line. Male fertility was defined as the percentage of red-eyed adults of the total offspring yield ((WT · adults^−1^) %) (Camus et al., 2017a).

### Larval development

Experimental flies were placed in separate oviposition chambers to mate and lay eggs for 2 hr. Eggs were then gently collected and placed in separate vials at a concentration of 30 eggs per vial. All vials were screened for newly eclosed adults three times daily (10 am, 1 pm, 4 pm) for a period of 14 days. This gave ample time for all developing flies to eclose, with any remaining pupae deemed dead. Both development time (hours) and sex were recorded. Survival to adulthood was also measured as the percentage of successfully hatched adults in each vial ((*n* adults · *n* eggs^−1^) %) (Rodríguez et al., 2021).

### Thermal tolerance

Heat tolerance assays involved exposing nonvirgin flies to a 39 °C environment (glass vials immersed in a circulating water bath) and recording the time (min) taken for each fly to succumb to heat stress (heat knock-down) (Camus et al., 2017b; Hoffmann et al., 2002). Cold tolerance assays involved exposing nonvirgin flies to a 0 °C environment (plastic tubes immersed in an ice-slurry water bath) for 4 hr to induce chill coma response. Tubes were then placed at 25 °C, and the time (min) taken for each fly to regain consciousness (standing upright) was recorded (chill-coma recovery time; CCRT). Cold tolerance was expressed as 120 minus CCRT (Camus et al., 2017b).

### Data analysis

Data were analyzed with the software R (R Core Team, 2021) and several supporting packages (see Supporting Information). Respirometry data was further analyzed using principal components analysis. Metabolic continuous variables were standardized prior analysis, and principal components were then extracted and analyzed as single parameters. A linear mixed model was implemented for each parameter, considering mitochondrial haplotype (“*mtDNA*”), nuclear background (“*nDNA*”) and sex (“*sex*”) as categorical fixed effects, as well as their two- and three-way interactions. Models accounted for differences in population replicate (“*pop*”), generational sampling block (“*batch*”), fly age (“*day*”), trial (“*run*”) and vial (“*vial*”), which were included as random effects. A generalized linear mixed model fitting the same fixed and random effects was implemented for traits following either Poisson or binomial distribution. Overdispersion was accounted for by adding an observation-level random effect to the mixed model. The significance of the three main factors and their possible interactions were determined through a type III ANOVA, followed by post hoc multi-comparison with adjustment for multiple testing. The best-fitting model was determined through a step-wise simplification by backward elimination of nonsignificant highest-order effects. The effect size was calculated as partial eta squared values ($\eta_{p}^{2}$). Supporting data are provided in Supplementary Tables S1–S11. Detailed procedures, protocols, and R packages used are provided in Supplementary Material.

## Results

### Mitochondrial phenotype

**Figure 1.**
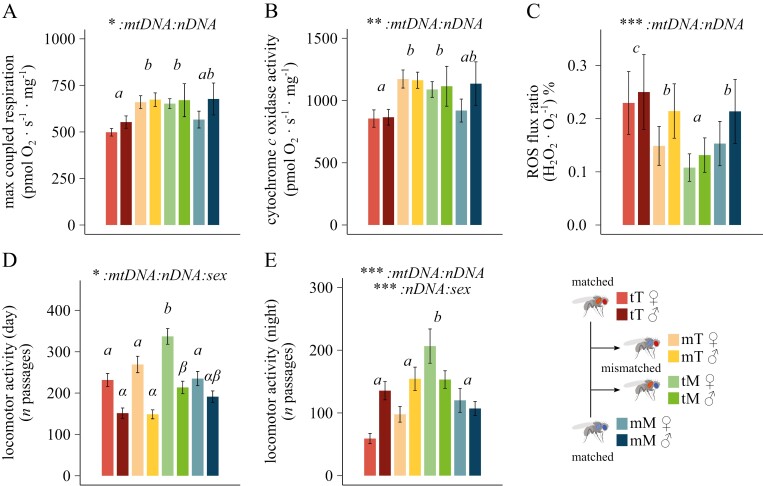
Mitochondrial phenotype and locomotor activity. (A and B) Mitochondrial respiration in permeabilized fly thoraces (pmol O_2_ · s^−1^ · mg^−1^) reflecting the *(a)* max coupled respiration sustained by CI, CII, ProDH, and GpDH-linked substrates, and *(b)* cytochrome *c* oxidase standalone capacity (*n* = 6). (C) Hydrogen peroxide production over oxygen consumption during max coupled respiration ((H_2_O_2_ · O_2_^−1^) %) (*n* = 6). (D and E) Fly locomotor activity during *(d)* daytime and *(e)* night (*n* passages) (*n* = 62–64). Statistical analyses: linear mixed model; Fixed effects: “*mtDNA*,” “*nDNA*” and “*sex*,” plus their interactions. Significance was determined by means of a type III ANOVA. Letters indicate statistical differences following a post hoc multi-comparison test. Comparison in (D) was run separately for each sex. Data are shown as mean ± sem. *p ≤ 0.05; **p ≤ 0.01; ***p ≤ 0.001. A detailed summary is reported in Supplementary Tables S1, S3, and S4.

The impact of mitonuclear interactions was tested at the level of mitochondrial respiration (O_2_ fluxes normalized for tissue mass—pmol O_2_ ∙ s^−1^ ∙ mg^−1^) sustained by different combinations of substrates. An interaction effect between the mitochondrial and the nuclear genomes was revealed for the max coupled (state 3) respiration (CI + ProDH + CII + GpDH_P_, *F* = 6.59, *p* = 0.014; $\eta_{p}^{2}$ = 0.15), max uncoupled (state 3u) respiration (CI + ProDH + CII + GpDH_E_, *F* = 4.66, *p* = 0.037; $\eta_{p}^{2}$ = 0.11), and cytochrome *c* oxidase standalone capacity (CIV_E_, *F* = 9.12, *P* = 0.0044; $\eta_{p}^{2}$ = 0.19) (Figure 1A and B; Supplementary Figure S2A; Supplementary Table S1). Overall, these results indicate that mitonuclear interactions can have an impact on mitochondrial respiration across both sexes, with mismatched cybrids (“mT,” “tM”) showing significantly increased respiratory rates compared to the matched population “tT,” as well as a trend of increased respiration with respect to the other matched population “mM” (Supplementary Table S1). These results were also supported by the analysis of the principal components (Supplementary Figure S3; Supplementary Table S1), where an interaction effect between mtDNA and nDNA reflecting the mismatched-specific mitochondrial phenotype was found for PC1 (*F* = 5.74, *p* = 0.02; $\eta_{p}^{2}$ = 0.13), with parameters “max coupled respiration,” “max uncoupled respiration” and ‘CIV capacity’ loading strongly on this axis (Supplementary Figure S3C and E). Variations in respiratory rates among mitonuclear lines were not associated with changes in tissue mass and mtDNA content. Thorax weight was influenced by sex (*F* = 77.72, *p* < 0.001; $\eta_{p}^{2}$ = 0.64), while mtDNA content by a nDNA by sex interaction (*F* = 6.73, *p* = 0.011; $\eta_{p}^{2}$ = 0.09) (Supplementary Figure S2B and C; Supplementary Tables S1 and S2), suggesting that males have lower body mass and higher mtDNA content than females, the latter only in lines with Townsville nuclear background (“tT,” “mT”).

The analysis of FCR (i.e., qualitative analysis with parameters normalized for their own max uncoupled respiration), as well as flux control factors, revealed no overall differences in substrate preferences dictated by mitonuclear combination (Supplementary Figure S4; Supplementary Table S1). That said, FCR CIV_E_ (Supplementary Figure S4A) and CIV excess capacity (j_ExCIV_) (Supplementary Figure S4B) were influenced by mitonuclear combination, with ‘mT’ population showing a trend of higher activity compared to all other populations. A main effect of sex was revealed for CI_P_ (*F* = 4.51, *p* = 0.039; $\eta_{p}^{2}$ = 0.1), CI + ProDH_P_ (*F* = 5.97, *p* = 0.018; $\eta_{p}^{2}$ = 0.12) and CI + ProDH + CII_P_ (*F* = 8.81, *p* = 0.0049; $\eta_{p}^{2}$ = 0.17) expressed as FCR (Supplementary Figure S4A), for CI_L_ (*F* = 10.57, *p* = 0.0022; $\eta_{p}^{2}$ = 0.20), CI + ProDH_P_ (*F* = 4.57, *p* = 0.039; $\eta_{p}^{2}$ = 0.12) and CI + ProDH + CII_P_ (*F* = 6.26, *p* = 0.017; $\eta_{p}^{2}$ = 0.16) expressed as O_2_ fluxes (Supplementary Figure S2A), as well as for PC2, with mitochondrial coupled respiration linked with CI, CI + ProDH and CI + ProDH + CII activity loading strongly on it (*F* = 4.52, *p* = 0.039; $\eta_{p}^{2}$ = 0.10) (Supplementary Figure S3D and F). Differences dictated by sex were also revealed for G3P_CF_ (*F* = 8.81, *p* = 0.0049; $\eta_{p}^{2}$ = 0.17), reflecting the increase in respiration following glycerophosphate addition (Supplementary Figure S4B). Overall, these results suggest sex-specific differences in substrate preference, with males having higher respiratory rates sustained by CI, ProDH, and CII complexes, while females rely more on GpDH activity to sustain maximal state 3 respiration.

ROS production rate (pmol H_2_O_2_ ∙ s^−1^ ∙ mg^−1^) was measured in parallel with mitochondrial respiration (Supplementary Table S3). Differences among sexes were revealed in ROS fluxes during max coupled respiration and during total inhibition of the ETS (maximal ROS production) (*F* = 10.16, *p* = 0.0028, $\eta_{p}^{2}$ = 0.21, and *F* = 31, *p* < 0.001, $\eta_{p}^{2}$ = 0.43, respectively), with males having generally higher ROS production rates compared to females (Supplementary Figure S5A and B). When further scrutinizing ROS efflux rate over the concomitant respiratory rates (H_2_O_2_ · O_2_^−1^), we, however, found a significant mtDNA by nDNA interaction during max coupled respiration (*F* = 13.18, *p* < 0.001; $\eta_{p}^{2}$ = 0.27), indicating lower ROS production per molecule of oxygen consumed in mismatched populations (“mT,” “tM”) compared to their genetically closest (at the level of nuclear background) matched populations (“tT,” “mM”) (Supplementary Figure 1C; Supplementary Table S3). The max ROS ratio (inhibited ETS) was mainly influenced by both nDNA and sex, without interaction (Supplementary Figure S5C).

### Life history traits

In addition to mitochondrial physiology, the impact of mitonuclear interactions was further tested on different life history traits. An interaction effect between the mitochondrial and the nuclear genome was found for fly locomotor activity during the day, which also varied across sexes (*F* = 5.02, *p* = 0.025; $\eta_{p}^{2}$ = 0.01), and during the night (*F* = 21.27, *p* < 0.001; $\eta_{p}^{2}$ = 0.04) (Figure 1D and E, Supplementary Figure S6; Supplementary Table S4). In line with the trend in respirometry results, “tM” flies are more active than both “tT” and “mM” matched flies during both day and night, whereas the activity of “mT” individuals does not significantly differ. That said, a higher night locomotor activity of mismatched individuals compared with individuals from the genetically closest matched population is supported when comparing lines within a common nuclear background (“tT”-“mT” and “tM”-“mM”) (Supplementary Table S4).

**Figure 2.**
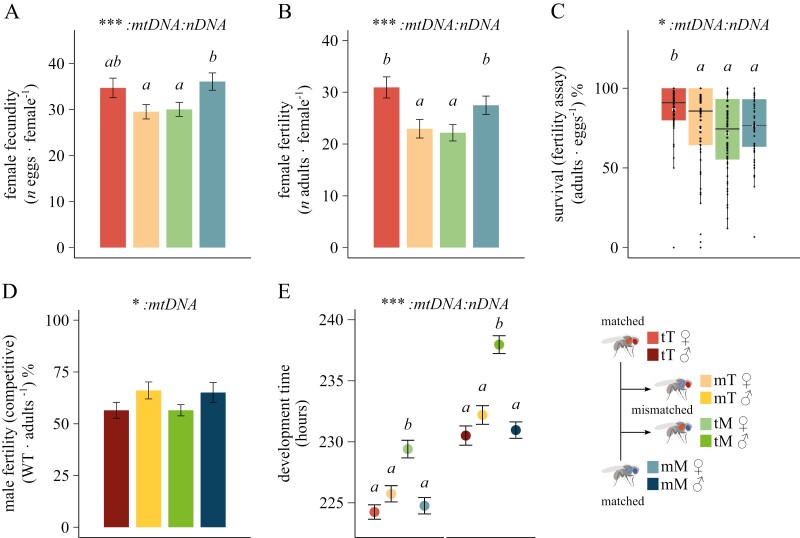
Life history traits. (A–C) Female reproductive success is expressed by *(a)* the number of eggs laid by focal females mated with standardized LHm males (fecundity—*n* eggs · female^−1^), *(b)* the number of adult offspring produced for each lay (fertility—*n* adults · female^−1^), and *(c)* percentage of adults survival in each vial ((adults · eggs^−1^) %) (*n* = 56–60). (D) Male reproductive success expressed as the percentage of wild-type individuals over the total number of offspring—fertility (fertility—*n* WT · adults^−1^) (*n* = 20). (E) Developmental fitness expressed by the egg-adult development time (hours) (*n* = 177–280). Statistical analyses: (A, B, C, and D) generalized linear mixed model; (E) linear mixed model. Fixed effects: “*mtDNA*,” “*nDNA*,” and “*sex*” (only for E), plus their interactions. Significance was determined by means of a type III ANOVA. Letters indicate statistical differences following a post hoc multi-comparison test. Data (A, B, D, and E) shown as mean ± sem. *p ≤ 0.05; **p ≤ 0.01; ***p ≤ 0.001. A detailed summary is reported in supplementary tables s5, s7, s8.

Mitonuclear interactions were also found to have a pervasive effect on reproductive success and offspring development. Mitonuclear interactions impacted fecundity (*X*^2^ = 11.13, *p* < 0.001), female fertility (*X*^2^ = 16.50, *p* < 0.001), and survival (*X*^2^ = 5.95, *p* = 0.0147), with mismatched females having less offspring compared with females from both matched populations (Supplementary Figure 2B; Supplementary Table S5). Additionally, mismatched lines laid fewer eggs (Figure 2A) compared to matched lines in which they have the nuclear genome in common (i.e., “tT” vs “mT” and “mM” vs “tM” (Supplementary Table S5—comparisons run within each nuclear background), and displayed reduced egg to adult survival (Figure 2C) compared to the matched ‘tT’ line. Differences dictated by the nuclear genotype were revealed for male fertility in a noncompetitive environment (*X*^2^ = 5.34, *p* = 0.021), with lines bearing the “M” nuclear genomes having fewer offspring than lines with ‘T’ nuclear background (Supplementary Figure S7B; Supplementary Table S6). On the other hand, male fertility in a competitive environment was influenced by the mtDNA (*X*^2^ = 6.49, *p* = 0.011), with the “m” haplotype associating with higher fertility than the ‘t’ one (Figure 2D; Supplementary Table S7). Larval developmental time was also influenced by the mitonuclear combination (*F* = 58.21, *p* < 0.001; $\eta_{p}^{2}$ = 0.03), with female and male individuals of the mismatched “tM” mitonuclear line developing slower than flies from both matched populations (Figure 2E; Supplementary Table S8). Although individuals from “mT” mismatched line only display a trend of slower developmental rate compared to their closest matched population (“tT”), a significant difference between the two lines was revealed in females when testing the impact of mtDNA within each nuclear background and sex (Supplementary Table S8). The interaction between mtDNA and nDNA genomes additionally influenced survival during development (*X*^2^ = 4.34, *p* = 0.037), with individuals from matched “tT” populations showing signatures of better survival to adulthood compared with both mismatched populations (“mT” and “tM”) and the Melbourne matched line (“mM”) (Supplementary Figure S7A; Supplementary Table S9).

### Thermal tolerance

**Figure 3.**
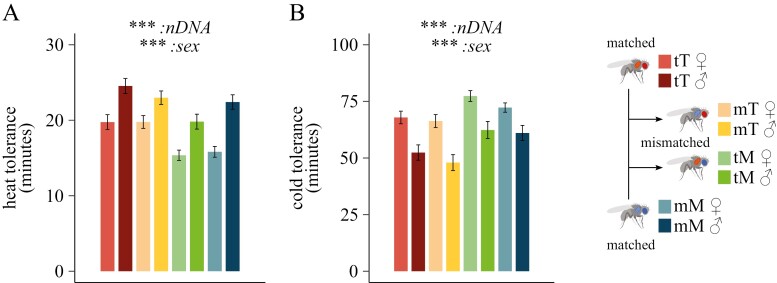
Thermal tolerance. (A) Heat shock tolerance is measured as the time (min) taken for each fly to enter a coma-like state following 39 °C heat shock (*n* = 50). (B) Cold shock tolerance was expressed as 120 minus the time (min) taken for each fly to regain consciousness after chill-induced coma (*n* = 60). Statistical analyses: linear mixed model; Fixed effects: “*mtDNA*,” “*nDNA*,” and “*sex*,” plus their interactions. Significance was determined by means of a type III ANOVA. Data are shown as mean ± sem. *p ≤ 0.05; **p ≤ 0.01; ***p ≤ 0.001. A detailed summary is reported in Supplementary Tables S10–S11.

We finally assessed the influence of nuclear and mitonuclear genomes on fly thermal performance. Heat and cold tolerance parameters were influenced by the solely nDNA (*F* = 30.48, *p* < 0.001, $\eta_{p}^{2}$ = 0.07, and *F* = 19.87, *p* < 0.001, $\eta_{p}^{2}$ = 0.04, respectively) and sex (*F* = 56.56, *p* < 0.001, $\eta_{p}^{2}$ = 0.13, and *F* = 48.57, *p* < 0.001, $\eta_{p}^{2}$ = 0.09, respectively) (Supplementary Tables S10 and S11). In line with the natural latitudinal segregation of the two native populations, mitonuclear combinations with northern-derived Townsville nDNA (“tT,” “mT”) were more resistant to heat shock than flies bearing the southern-derived Melbourne nDNA (“tM,” “mM”) (Figure 3A). On the other hand, lines with Melbourne nDNA better recovered from cold shock than lines with Townsville nDNA (Figure 3B). Females and males also showed divergent thermal tolerance patterns, with female flies better able to withstand cold stress than males, whereas male flies were more resistant to heat stress than their female counterparts (Figure 3A and B; Supplementary Tables S10 and S11).

## Discussion

One of the many consequences of local adaptation is the divergence of genomic information. This information not only applies to the nuclear genome, but an accumulating number of studies have highlighted the role of the mitochondrial genome as an important source of adaptive variation (Blier et al., 2001; Dowling et al., 2008; Lane, 2009, 2011; Wolff et al., 2014). Here, we explored how mitonuclear epistasis shapes energy flow at the cellular level and how variation in aerobic metabolism might influence key evolutionary and ecological patterns. We observed higher respiratory rates in *Drosophila* mismatched cybrids compared with the population with matched “tT” genomes in both females and males (Figure 1A and B; Supplementary Figures 2A and S3). Additionally, we see a trend of increased oxygen consumption in mismatched lines when compared with the other matched population (mM). These results are linked with a decreased amount of ROS production per molecule of oxygen consumed in both mismatched cybrids compared to their genetically closest-matched population (Figure 1C). These differences were not underpinned by changes in thorax mass or mitochondrial content (Supplementary Figure S2B and C), nor with changes in substrate preferences, which could indicate a shift in metabolic flux to compensate for the genomic change (Supplementary Figure S4).

The genetic basis of the physiological differences observed is, however, uncertain. Previous research has described 15 synonymous SNPs differentiating the two haplotypes, with these SNPs being widespread among most coding genes (Camus et al., 2017b). Although traditionally considered functionally silent, evidence exists that synonymous mutations can be evolutionarily significant, impacting mRNA stability, translation speed, folding and posttranslational modifications of proteins, as well as enzyme structure and function (Hurst, 2011; Jiang et al., 2022; Shabalina et al., 2013). Moreover, studies highlighted codon usage bias in mtDNA, where codons encoding the same amino acid are not used interchangeably. This suggests that switching from one synonymous codon to another might not be completely silent (Wei et al., 2014; Yang & Nielsen, 2008). It is, therefore, possible, albeit untested, that the synonymous variation found between our two haplotypes might be driving the phenotypic effects. Alternatively, variation in the control region (D-loop) might underpin these effects (Hopkins et al., 2017; Rollins et al., 2016). The *D. melanogaster* control region is ~6kb long with AT-richness of >85%, making it hard to accurately sequence. Furthermore, alternative genes within the mitochondrial genome with potential impact on mitochondrial respiration have been identified in human mtDNA (Breton, 2021; Kienzle et al., 2023)—a mechanism that has not been investigated in the *Drosophila* mtDNA and could potentially be modulating our described phenotypes. In the case of our two main haplotypes (“t” and “m”), previous work has found differences in gene expression patterns when these haplotypes were coupled to a common isogenic nuclear background (Camus et al., 2017b). It is, therefore, possible (although yet tested in our massbred populations) that similar haplotype-mediated differences in mtDNA transcription might underpin the effects described in our study.

Hybridization can sometimes be beneficial, providing an effective source of adaptive alleles that overcome the fitness cost of mitonuclear incompatibility (Hill, 2019). This has been exemplified in both migrating birds and *Drosophila*, showing signs of adaptive mitonuclear co-introgression (Beck et al., 2015; Morales et al., 2018; Toews et al., 2014). That said, the general pattern expected from mitonuclear incompatibilities is a breakdown of mitochondrial efficiency (reduced OXPHOS capacity), associated with deleterious fitness consequences (Burton et al., 2006; Ellison & Burton, 2006; Ellison et al., 2008; Meiklejohn et al., 2013; Moran et al., 2024; Niehuis et al., 2008). Our findings also unmasked a phenotypic cost to mitonuclear mismatching. Despite the trend of increased respiration (Figure 1A and B), maintenance of ROS homeostasis (Figure 1C; Supplementary Figure S5) and locomotory performance (Figure 1D and E; Supplementary Figure S6) in mismatched populations, they were less fertile than matched populations. Conversely, from male fertility, which was impacted by the solely mitochondrial or nuclear genotype (Figure 2D; Supplementary Figure 7B), a pervasive mitonuclear interaction effect was revealed for all the traits associated with female reproduction, larvae development and survival. Females from mismatched populations had a reduced number of offspring (Figure 2B) compared with both matched populations, as well as decreased fecundity (Figure 2A) and larvae survival (Figure 2C; Supplementary Figure 7A) relative to one matched population (Supplementary Tables S5 and S9). Moreover, one of the mismatched population had slower larval development with respect to both matched ones (Figure 2E). Notably, when the impact of mitochondrial introgression was tested in each nuclear background separately (comparing rarer vs. predominant mitonuclear lines in each cline population), females of both cybrid lines showed decreased fecundity, fertility, and slower larval development compared to the matched population that shared the same nuclear background (Supplementary Tables S5 and S8).

Taken together, our findings suggest that the overall increase in respiratory rate of mismatched populations described here might reflect an overall dysregulation of mitochondrial function (e.g., via some form of overcompensation for deficient mitochondria (Moreno-Loshuertos et al., 2006; Sercel et al., 2024), rather than improved OXPHOS efficiency. This is in line with evidence suggesting that elevated metabolic rates might reflect physiological dysfunction in natural cybrids, contributing to the cost of hybridization (Bize et al., 2018; Chapdelaine et al., 2020; Combs et al., 1997; Gvoždík, 2012; Hoekstra et al., 2013; McFarlane et al., 2016).

Organisms must adopt life history strategies when allocating the finite energetic resources available, as increasing the energy allocation to one trait inevitably reduces the availability for the others (Chang et al., 2021). Examples of trade-offs between locomotory metabolic processes (locomotor activity or flight) and mainly biosynthetic ones (reproduction and growth) are widespread in the literature (Chang et al., 2021; Gibbs et al., 2010; Husak et al., 2016; Zhang et al., 2009). Although high metabolic rates generally correlate with fast development, early reproduction and decreased longevity (Pettersen et al., 2016), previous evidence in wild-type *Drosophila* lines linked high complex IV activity and metabolic rates with lowered fecundity and lifespan (Ballard et al., 2007a; Mołoń et al., 2020). Furthermore, mitonuclear interactions were found to have a substantial impact on resource allocation and life history trade-offs in flies (Camus et al., 2020b). Our results suggest the effects of mitonuclear interactions on *Drosophila* phenotype, involving a trade-off between mitochondrial bioenergetics and organismal fitness in mismatched populations.

The physiological basis of these life history dynamics is uncertain, as our measures of mitochondrial bioenergetics were obtained from thoraces (flight muscle), whose bioenergetic requirements are likely to be very different to tissues more heavily involved with reproduction. Compared with somatic tissues, gonads primarily need to power biosynthesis for gamete production, and the relative requirements for ATP synthesis are 10-fold lower than flight muscle (Camus et al., 2023; Wetzker & Reinhardt, 2019). The different metabolic profiles of somatic and reproductive tissues are also accompanied, at least in some mammals, by the expression of gamete-specific nuclear isoforms of some OXPHOS genes (Huttemann et al., 2003; Liu et al., 2006), opening up the opportunity for divergent mitonuclear coevolution (and incompatibilities) in somatic tissues and sex organs. Given these different energetic requirements between tissues, the trade-off in our mismatched flies might be explained by the way that mitochondria power biosynthesis rather than OXPHOS itself (Lane, 2022). At the level of gonads, this may result in the compromised fertility observed in mismatched lines, as well as underpinning the slower growth and decreased survival of their larvae. Reallocation of resources away from gamete production (and growth in general) could, in turn, explain the higher aerobic capacity of somatic tissues and locomotor activity in mismatched populations. Our future studies will aim to investigate whether the main phenotypic effect of mitonuclear mismatch might differ in gonads, potentially explaining the decrease in components of fitness.

We detected differences in thermal tolerance between our two genomic backgrounds, with northern and southern populations respectively, being more resistant to heat and cold stress (Figure 3). These results corroborate previous work on thermal tolerance differences in the Australian east-coast cline (Hoffmann & Weeks, 2007; Hoffmann et al., 2002; Sgro et al., 2010). Temperature is a well-known metabolic stressor, which could exacerbate and even drive the main effect of mitochondrial introgression (Rank et al., 2020). Our data highlight the nuclear genome as the main contributor to thermal tolerance, with the mtDNA having a much lower contribution than previously measured (Camus et al., 2017b; Lajbner et al., 2018). However, in these studies, the mtDNA variants were coupled to a standardized isogenic nuclear background plus had double the sample size (Camus et al., 2017b), allowing for a more precise control of genetic effects and higher power to detect small genetic effects. It is possible that mtDNA effects on thermal traits are more subtle and get swamped by the large genetic variance coming from the nuclear genome. Finally, we cannot exclude that adaptation to chronically increased temperature (or large temperature fluctuations) might have a mitonuclear contribution. Future research will, therefore, aim to test that.

Mitonuclear incongruences have been proposed as specific cases of Bateson–Dobzhansky–Muller incompatibilities (Burton et al., 2006). Depending on the severity of the incompatibility (either via high genetic divergence or large-effect SNPs), mitonuclear epistasis may restrict gene flow between populations and has the potential to reinforce reproductive isolation (Burton, 2022; Gershoni et al., 2009; Hill, 2019). Despite the absence of allopatry and the low mitochondrial genetic divergence between fly populations, we found evidence that rarer combinations of mtDNA haplotype and nuclear background are associated with decreased reproductive performance in females. These results support the existence of partial genetic barriers dictated by the mitonuclear combination. Consequently, mitonuclear coadaptation under climatic selection might contribute to the evolutionary trajectory, clinal distribution, and future ecological adaptation of natural fly populations in eastern Australia.

In this study, we tested the extent to which mitonuclear epistasis impacts organismal fitness in replicated populations of flies with different combinations of mtDNA and nuclear genotype. Our results suggest that even small differences in mitonuclear genotype can impair fertility, potentially reducing gene flow among fly populations adapted to different thermal niches. Curiously, these mitonuclear genotypes boosted respiratory outputs and locomotor activity in some mismatched lines, suggesting a possible trade-off between aerobic capacity and fertility, which might reflect limited metabolic plasticity in mitochondrial function. We live in a rapidly changing world in which natural populations are experiencing unprecedented changes in temperature, diet, and geographical distribution. If mitonuclear interactions constrain metabolic plasticity and fitness, then it will be important to include mitonuclear epistasis in ecological studies of the adaptive capacity of natural populations.

## Supplementary material

Supplementary material is available online at *Evolution Letters*.

qrae043_suppl_Supplementary_Materials

## Data Availability

Supportive information is provided as online supplementary text and tables. All data used in this study and the scripts used for the statistical analysis are available on figshare online repository: https://doi.org/10.6084/m9.figshare.24162879.v1.
